# Synthesis and Antibacterial Properties of Oligomeric Dehydrogenation Polymer from Lignin Precursors

**DOI:** 10.3390/molecules27051466

**Published:** 2022-02-22

**Authors:** Xin Wei, Sheng Cui, Yimin Xie

**Affiliations:** 1 Research Institute of Pulp and Paper Engineering, Hubei University of Technology, Wuhan 430068, China; wxpaper123@163.com (X.W.); cui2019hbut@163.com (S.C.); 2 Hubei Provincial Key Laboratory of Green Materials for Light Industry, Hubei University of Technology, Wuhan 430068, China

**Keywords:** lignin, dehydrogenation polymerisation, antibacterial property, oligomer, chemical structure

## Abstract

The lignin precursors of coniferin and syringin were synthesised, and guaiacyl-type and guaiacyl-syringyl-type oligomeric lignin dehydrogenation polymers (DHP and DHP-GS) were prepared with the bulk method. The carbon-13 nuclear magnetic resonance spectroscopy showed that both DHP-G and DHP-GS contained β-O-4, β-5, β-β, β-1, and 5-5 substructures. Extraction with petroleum ether, ether, ethanol, and acetone resulted in four fractions for each of DHP-G (C_11_–C_14_) and DHP-GS (C_21_–C_24_). The antibacterial experiments showed that the fractions with lower molecular weight had relatively strong antibacterial activity. The ether-soluble fractions (C_12_ of DHP-G and C_22_ of DHP-GS) had strong antibacterial activities against *E. coli* and *S. aureus*. The C_12_ and C_22_ fractions were further separated by preparative chromatography, and 10 bioactive compounds (G_1_–G_5_ and GS_1_–GS_5_) were obtained. The overall antibacterial activities of these 10 compounds was stronger against *E. coli* than *S. aureus*. Compounds G_1_, G_2_, G_3_, and GS_1_, which had the most significant antibacterial activities, contained β-5 substructures. Of these, G_1_ had the best antibacterial activity. Its inhibition zone diameter was 19.81 ± 0.82 mm, and the minimum inhibition concentration was 56.3 ± 6.20 μg/mL. Atmospheric pressure chemical ionisation mass spectrometry (APCI-MS) showed that the antibacterial activity of G_1_ was attributable to a phenylcoumarin dimer, while the introduction of syringyl units reduced antibacterial activity.

## 1. Introduction

Lignin is one of the most abundant natural materials in the world, accounting for about a quarter of wood tissue, and is therefore a promising renewable material [1,2,3]. This highly branched aromatic polymer is a natural macromolecular compound with an amorphous structure formed by dehydrogenation and polymerisation of three phenylpropane structural monomers: sinapyl alcohol, coniferyl alcohol, and *p*-coumaryl alcohol [4,5,6]. Lignin mainly exists in the cell walls of plants and has good antioxidant and antibacterial properties [7,8,9,10,11]. Its antibacterial properties help to reduce the risk of bacterial colonisation on the surface of materials [12]. Lignin and its derivatives are considered good candidate materials for medicine and health care.

Domínguez-Robles et al. [13] prepared a composite of softwood kraft lignin and polybutylene succinate (PBS) that was resistant to the adhesion of *S. aureus*, achieving a reduction in bacterial adhesion of approximately 90% compared to PBS. Kaur et al. [14] chemically modified bagasse lignin through acetylation, epoxidation, and hydroxymethylation reactions. They found that among the modified lignin samples, epoxy lignin had the most effective antibacterial activity, with minimum inhibitory concentrations (MIC) against *Bacillus aryabhattai* and *Klebsiella* of 90 and 200 µg/mL, respectively, demonstrating that lignin has great potential for antibacterial applications. Moreover, the structure and biological activity of lignin are greatly affected by the separation and extraction methods used as well as the molecular weight of the obtained product [15,16,17]. Furthermore, Rocca et al. [18] synthesised lignin-doped silver and gold nanoparticles by one-pot thermochemical and photochemical methods, and they found that the nanoparticles had a certain inhibitory effect on *E. coli* and *S. aureus*. More importantly, the particles are non-cytotoxic towards human cells at the bactericidal concentrations. Marulasiddeshwara et al. [19] also found that lignin capped silver nanoparticles (LCSN) not only have antioxidant and antibacterial properties, but also did not lyse red blood cell (RBC) membrane when assayed hemolytic activity suggested its non-toxic nature. Lourencon et al. [20] used eucalypt kraft lignin fractionated at pHs 9, 7, 5, and 3 by sequential acid precipitation. Fractions precipitated at pHs 9 and 7 have shown an outstanding antibacterial activity against five bacteria. Moreover, fractions 7 and 5 presented at cytotoxicity tests ability to inhibit the growth of U87MG and T98G glioma cells, while only a slight inhibition of adult human fibroblasts was detected. There studies show that lignin has a broad application prospect biological activity.

Enzymatically-synthesised dehydrogenation polymer is one of the most widely accepted lignin model compounds and the best lignin substitute used in various experiments. Its properties are highly similar to natural lignin [21]. In the process of synthesising DHP, the reaction conditions can be artificially controlled to adjust the type of DHP produced [22,23]. Therefore, DHP has a low degree of polymerisation, simple structure, and connectivity, and more functional groups can be obtained, which can achieve higher biological activity than natural lignin [24,25,26].

Ye et al. [27] synthesised DHP with isoeugenol as a monomer and found that β-O-4, β-β, β-5, and β-1 were the main structures of DHP. Its structure was similar to natural lignin. Chen et al. [28] also used isoeugenol to synthesise DHP and found that the antioxidant activity IC_50_ of the ether soluble component was 0.12 g/L. However, these studies focus on the direction of the chemical structure rather than the biological activity, and there are structural differences between isoeugenol and coniferin [29,30,31]. Therefore, it is more representative to synthesise oligomeric DHP with the lignin monomer coniferin, syringin, and *p*-coumaryl alcohol glucoside as precursors.

To investigate the antibacterial activity of oligomeric DHP, coniferin and syringin were used as raw materials under β-glucosidase and laccase catalysis to synthesise G-type lignin dehydrogenation polymer (DHP-G) and GS-type lignin dehydrogenation polymer (DHP-GS). The DHPs were extracted with different organic solvents and their growth inhibition effects on *E. coli* and *S. aureus* were determined by the filter paper agar diffusion method. The ether extractives C_12_ and C_22_, which had high antibacterial activity, were further screened and purified by preparative chromatography. The obtained compounds G_1_, G_2_, G_3_, and GS_1_ were structurally identified by atmospheric pressure chemical ionisation mass spectrometry (APCI-MS). The source of their biological activity was discussed by analysing the relationship between structure and antibacterial effect.

## 2. Results and Discussion

### 2.1. ^13^C-NMR Spectral Analysis of DHPs

**Figure 1:**
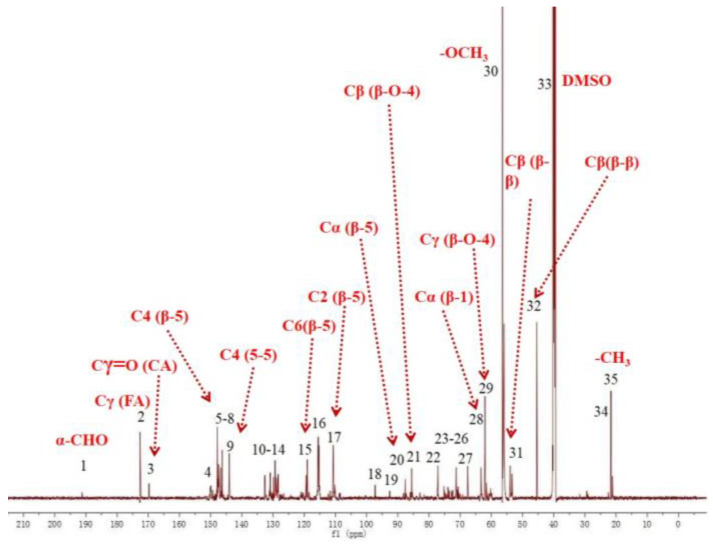
^13^C-NMR spectrum of DHP-G.

The ^13^C-NMR spectrum of DHP-G is shown in Figure 1. The weak signal peak at 190.9 ppm (No. 1) is an α-CHO produced by some oxidation during DHP polymerisation [32]. At 172.2 ppm (No. 2), the signal indicates oxidation at the γ-position to form cinnamic acid [33,34]. The signals at 149.8 to 147.0 ppm (No. 4–8) are attributable to the carbon atoms on the guaiacyl aromatic ring, and the signal at 143.6 ppm (No. 9) comes from the C4 of the 5-5 structure. The signals near 130 ppm (No. 10–14) indicate Cα/Cβ from C=C produced by the double bond on the side chain of coniferin and syringin. The signal at 87.2 (No. 20) is the Cα from the β-5 structure [35,36,37,38]. The signals at 67.2 ppm (No. 27) is the Cγ from the β-5 structure [22]. The signals at 70.2 ppm (No. 26), 85.1 ppm (No. 21), and 62.0 ppm (No. 29) are from the Cα, Cβ, and Cγ of the β-O-4 structure [39]. The signal at 63.5 ppm (No. 28) is from the Cα of the β-1 structure. The signals at 53.5 ppm (No. 31) and 46.0 ppm (No. 32) are attributed to the Cβ of the β-β structure [35,40]. These results indicate that DHP-G mainly includes β-5 and β-O-4 structures, but also includes some 5-5, β-1, and β-β structures.

**Figure 2:**
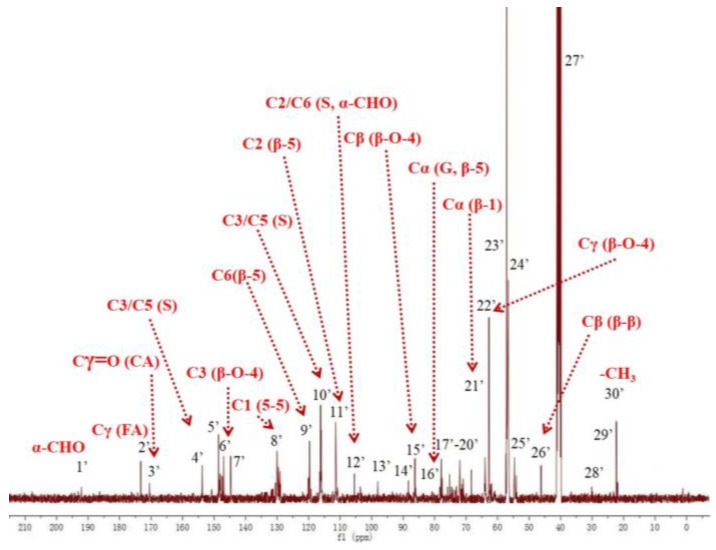
^13^C-NMR spectrum of DHP-GS.

The ^13^C-NMR spectrum of DHP-GS in Figure 2 is similar to the ^13^C-NMR spectrum of DHP-G in Figure 1, both of which are based on β-5 and β-O-4 structures while also including 5-5, β-1, and β-β structures. The difference is that DHP-GS contains some signals generated by syringyl units. For example, the signal at 152.8 ppm (No. 4′) comes from C3 and C5 on etherified syringyl units, while DHP-G does not have these structures [41,42].

### 2.2. Molecular Weight Analysis of DHP

The average molecular weights of the DHP fractions are shown in Table 1. The molecular weights of the DHP fractions increase with the enhancement of solubility of the solvent. Based on the molecular weight of coniferyl alcohol (180 Da) and the molecular weight of sinapyl alcohol (210 Da), the average molecular weights of the petroleum ether fractions C_11_ and C_21_ are 289 and 293 Da, respectively. These values are lower compared to coniferyl and sinapyl alcohol dimer, indicating that there were more monomer structures in the petroleum ether fractions.

### 2.3. Analysis of Antibacterial Properties of DHP Fractions

**Figure 3:**
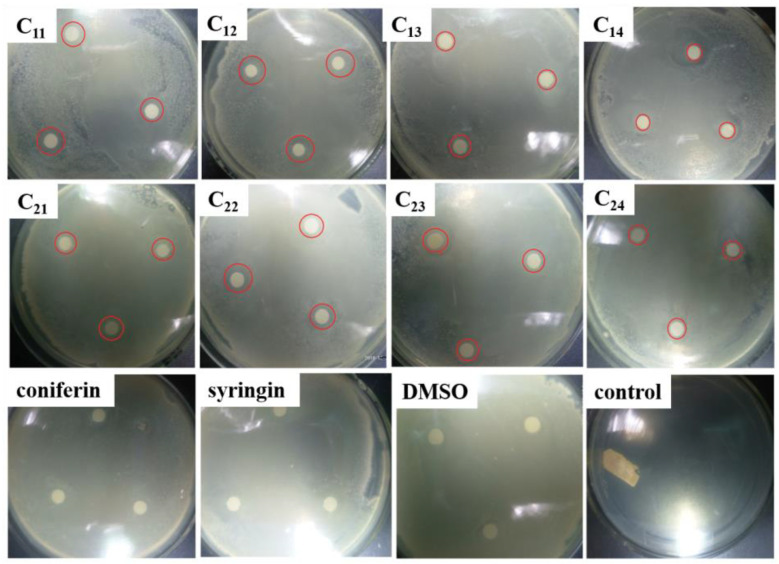
Effect of DHP fractions on growth inhibition of *E. coli*. Legend: The red circle area is the inhibition zone; coniferin and syringin: precursors of DHP; DMSO: No sample was added, only DMSO was added; control: control group without sample and solvent.

**Figure 4:**
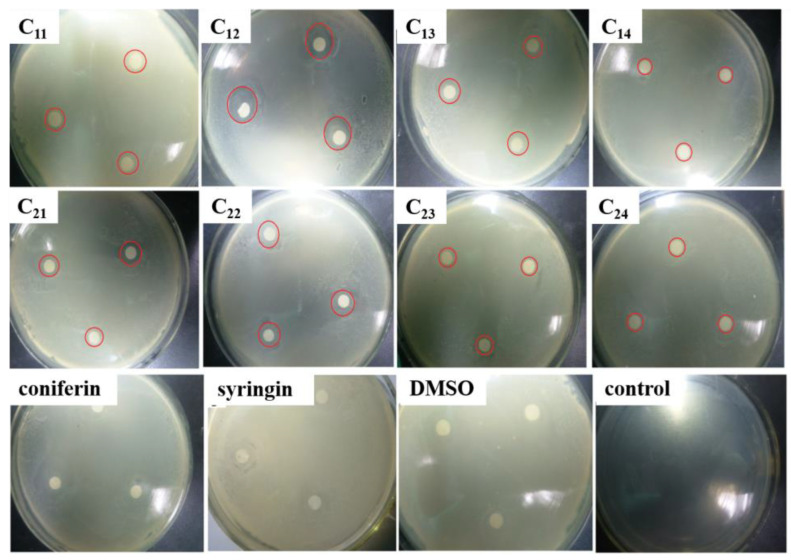
Inhibitory effect of DHP fractions on the growth of *S. aureus*. Legend: The red circle area is the inhibition zone; coniferin and syringin: precursors of DHP; DMSO: No sample was added, only DMSO was added; control: control group without sample and solvent.

**Figure 5:**
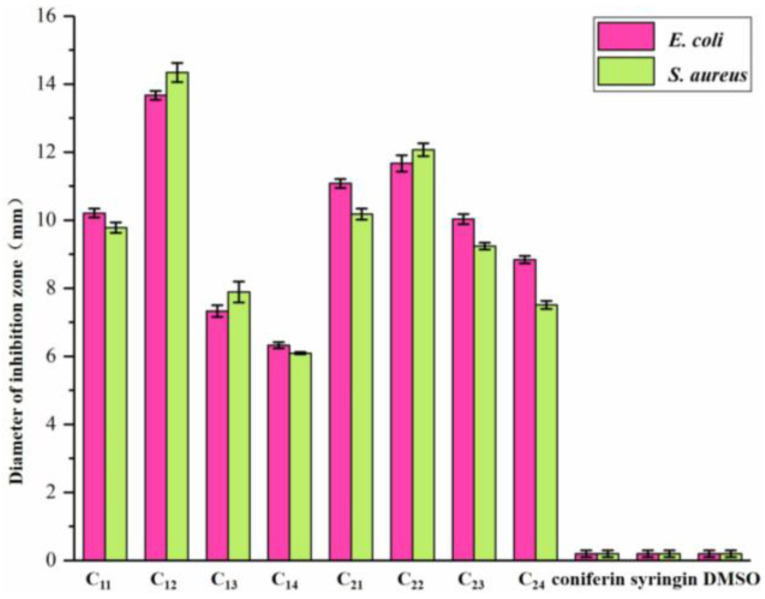
Inhibition zone diameter of DHP fractions on *E. coli* and *S. aureus*.

**Figure 6:**
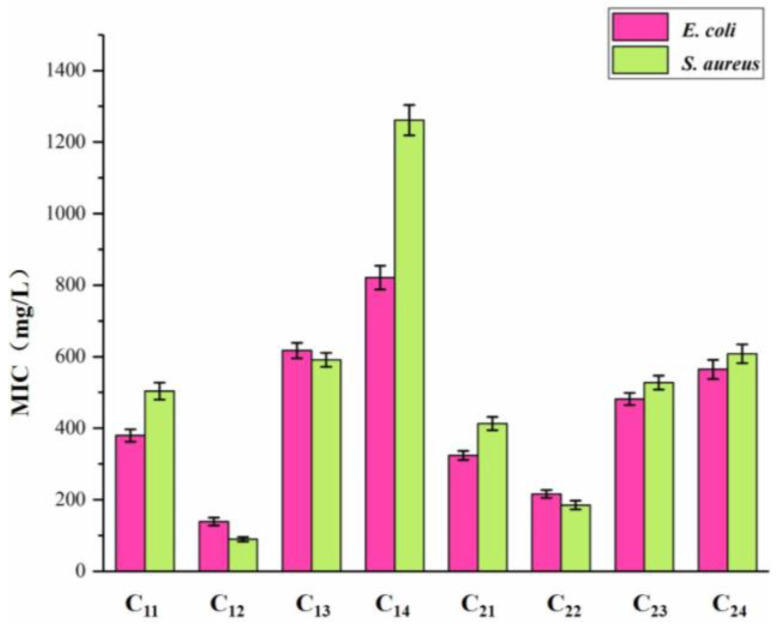
MIC of DHP fractions against *E. coli* and *S. aureus*. Legend: MIC: the minimum inhibitory concentration.

The inhibitory effects of DHP fractions, DHP precursors, and sample solvents on the growth of *E. coli* and *S. aureus* are shown in Figure 3 and Figure 4. The antibacterial effect of the sample was evaluated by the diameter of the inhibition zone, which is shown in Figure 5. While DHP precursor and sample solvent had no obvious inhibitory effect on the growth of the two tested bacteria, all DHP fractions had different inhibitory effects on the two tested bacteria. The petroleum ether-extracted fraction and the ether-extracted fraction, which had lower molecular weights, had better growth inhibition effects on the two tested bacteria than the ethanol-extracted and acetone-extracted fractions, indicating that the molecular weight may have a certain impact on the antibacterial performance of DHP. The ether-extracted fraction had the most obvious effect on the two tested bacteria. The diameters of the inhibition zone of the ether-extracted fraction of DHP-G on *E. coli* and *S. aureus* were 13.67 ± 0.21 mm and 14.34 ± 0.28 mm, respectively, compared to 11.67 ± 0.24 mm and 12.07 ± 0.19 mm for the ether-extracted fraction of DHP-GS. The minimum inhibitory concentration (MIC) of each fraction of DHP for the two tested bacteria is shown in Figure 6. The effect of the ether-extracted fraction on the two tested bacteria was the most obvious, as the MICs of the ether-extracted fraction C_12_ of DHP-G for *E. coli* and *S. aureus* were 138.90 ± 10.70 mg/L and 89.40 ± 6.50 mg/L, respectively. The MICs of the ether-extracted fraction C_22_ of DHP-GS for *E. coli* and *S. aureus* were 216.20 ± 11.30 mg/L and 185.10 ± 12.40 mg/L, respectively.

### 2.4. Analysis of Antibacterial Properties of Purified Components of DHP

**Figure 7:**
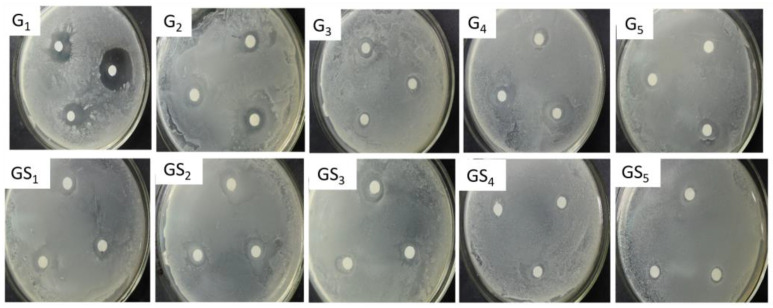
Inhibitory effect of purified compounds from DHP on the growth of *E. coli*. Legend: G_1_–G_5_: compounds purified from the ether fraction of DHP-G by preparative chromatography; GS_1_–GS_5_: compounds purified from the ether fraction of DHP-GS by preparative chromatography.

**Figure 8:**
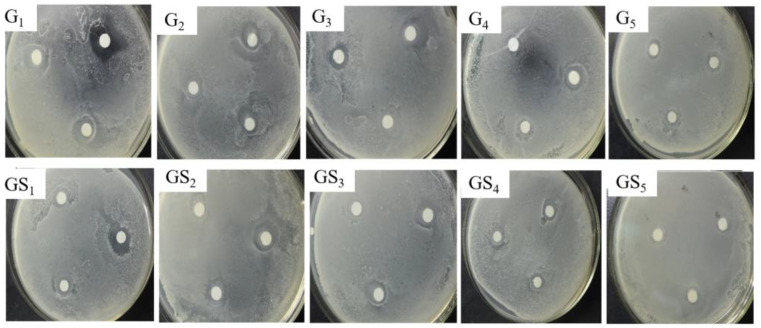
Inhibitory effect of purified components from DHP on the growth of *S. aureus*. Legend: G_1_–G_5_: compounds purified from the ether fraction of DHP-G by preparative chromatography; GS_1_–GS_5_: compounds purified from the ether fraction of DHP-GS by preparative chromatography.

**Figure 9:**
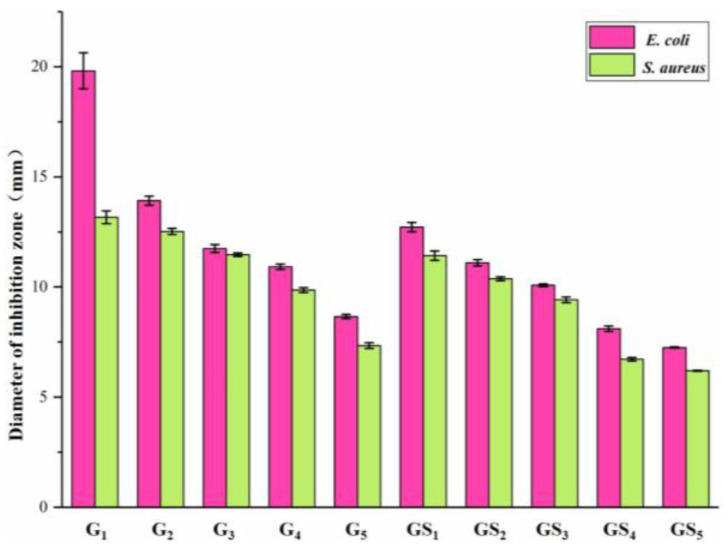
Inhibition zone diameter of purified compounds from DHP on *E. coli* and *S. aureus*.

**Figure 10:**
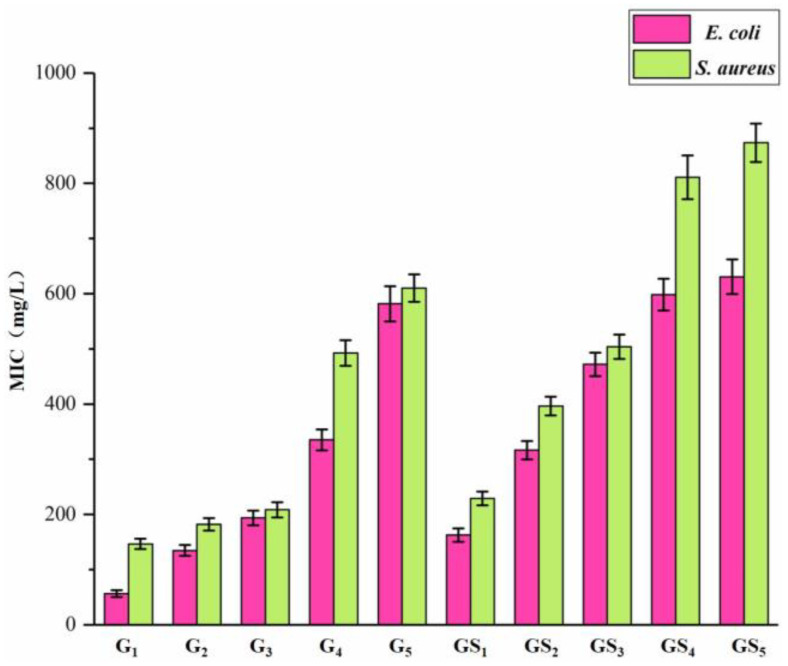
MIC of purified compounds from DHP against *E. coli* and *S. aureus*. Legend: MIC: the minimum inhibitory concentration.

The inhibitory effects of purified DHP compounds on the growth of *E. coli* and *S. aureus* are shown in Figure 7 and Figure 8. The diameter of the inhibition zone is shown in Figure 9. The MICs are shown in Figure 10. In general, the antibacterial activities of purified compounds of the ether fractions C_12_ and C_22_ (G_1_–G_5_ and GS_1_–GS_5_) were stronger against *E. coli* than against *S. aureus*. Among the purified compounds of DHP-G, G_1_ had the best inhibitory effect on the two tested bacteria. The diameters of the inhibition zone for *E. coli* and *S. aureus* were 19.81 ± 0.82 mm and 13.16 ± 0.29 mm, respectively, while the MICs were 56.30 ± 6.20 μg/mL and 146.50 ± 9.40 μg/mL, respectively. Among the purified compounds of DHP-GS, the compound GS_1_ had the best inhibitory effect on the two tested bacteria, and the diameters of the inhibition zone for *E. coli* and *S. aureus* were 12.72 ± 0.21 mm and 11.42 ± 0.21 mm, respectively, with MICs of 162.50 ± 12.20 μg/mL and 229.00 ± 12.50 μg/mL, respectively.

### 2.5. Structural Analysis of Bioactive Purified DHP Compounds by Mass Spectrometry

**Figure 11:**
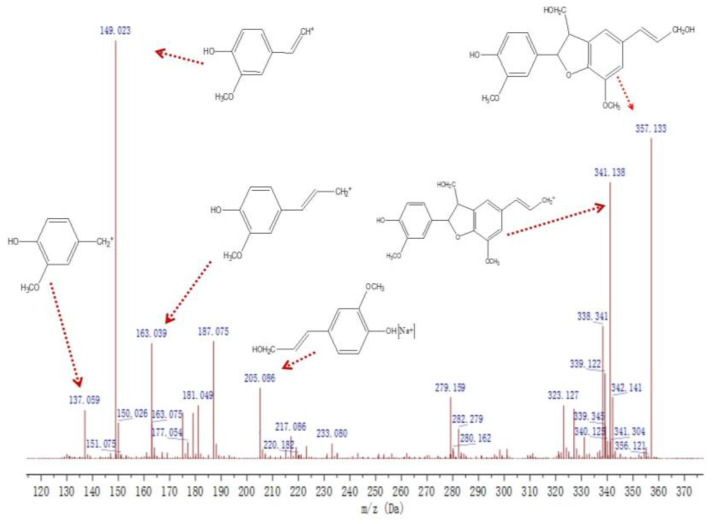
Mass spectrum of the compound G_1_.

The mass spectral information of G_1_ is shown in Figure 11. The ionisation of the γ-position carbon in the side chain of the coniferyl alcohol monomer formed a fragment peak at *m*/*z* 149.023, while a fragment peak was formed by the cleavage of the γ-hydroxyl at *m*/*z* 163.039. The ion signal peak at *m*/*z* 279.159 was derived from the dimer of the β-5 structure. The fragment peak at *m*/*z* 341.138 came from β-5, γ-CH_2_^+^. From the analysis of the fragment peaks, it was found that the G1 molecular ion at *m*/*z* 357.133 was a G-type dimer (β-5, γ-CH_2_OH, γ′-CH_2_OH).

**Figure 12:**
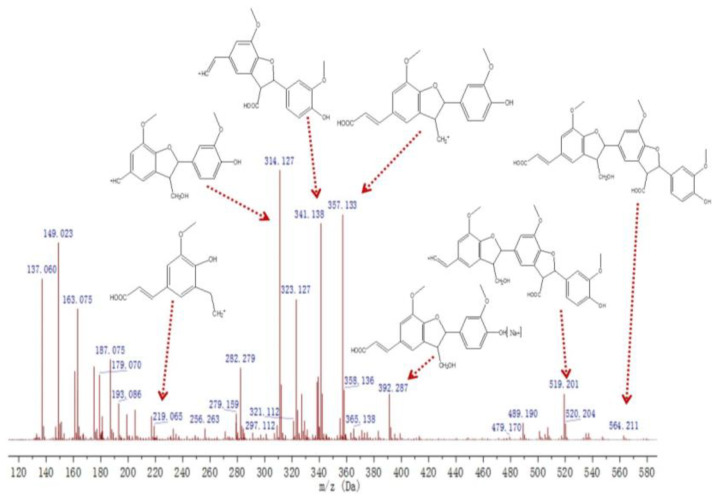
Mass spectrum of the compound G_2_.

The mass spectral information of G_2_ is shown in Figure 12. The signal at *m*/*z* 219.065 was derived from the fragmentation peak formed by the ether bond and carbon–carbon bond breakage of the coumaran ring in the phenylcoumaran structure. The signal at *m*/*z* 314.177 was from the cleavage of the carbon–carbon double bond in the side chain of the β-5 dimer. The peak at *m*/*z* 341.138 came from β-5 with γ-COOH, which acted as the precursor ion for the fragment at *m*/*z* 219.065. The signal at *m*/*z* 392.287 was generated by the capture of Na^+^ by the β-5 (γ-CHOH, γ′-COOH) dimer, indicating that many monomers are polymerised through the β-5 bond, and that the side chain easily oxidised into a carboxylic acid. The molecular ion peak of G_2_ appeared at *m*/*z* 564.221. According to the fragment peak analysis, the structure of G_2_ is most likely a G-type trimer ((β-5)(β-5), γ-COOH, γ′-CH_2_OH, γ″-COOH). The signal peak at *m*/*z* 519.201 was derived from the trimer ((β-5)(β-5), γ-COOH, γ′-CH_2_OH, β″-CH^+^). The loss of the carboxyl group at the γ position also confirmed the structure of G_2_.

**Figure 13:**
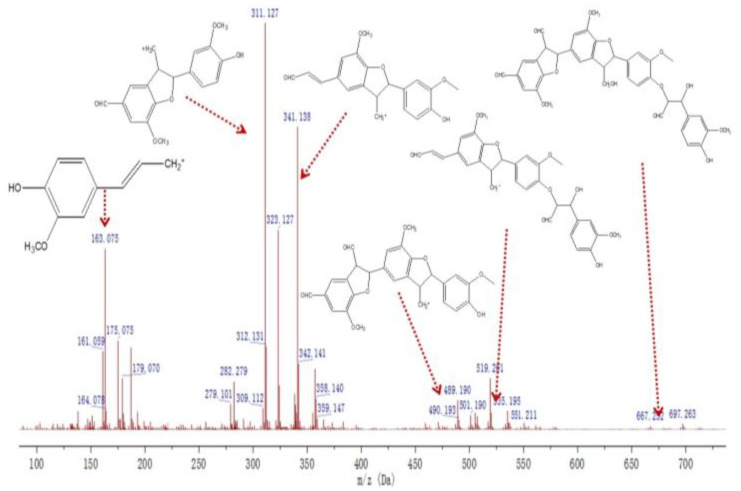
Mass spectrum of the compound G_3_.

The mass spectral information of G_3_ is shown in Figure 13. The signal peak at *m*/*z* 163.075 originated from the cleavage of the alcohol hydroxyl group at the γ position of the coniferyl alcohol monomer. The signal at *m*/*z* 282.279 was weak and represented the signal formed by the re-fracture of the ((β-5), α-CHO) structure at *m*/*z* 311.127. The peak at *m*/*z* 490.193 originated from the cleavage of the alcoholic hydroxyl group at the γ position of the ((β-5)(β-5), γ-CH_2_OH, γ′-CHO, α′-CHO) structure. The peaks at *m*/*z* 535.195 and *m*/*z* 551.211 are both attributed to the ((β-O-4)(β-5), α-OH, γ-CHO, γ′-CH_2_OH, γ″-CHO) structure. According to the fragment peak analysis, the G_3_ molecular ion peak at *m*/*z* 697.263 represents a tetramer ((β-O-4)(β-5)(β-5), α-OH, γ-CHO, γ′-CH_2_OH, γ″-CHO, α‴-CHO).

**Figure 14:**
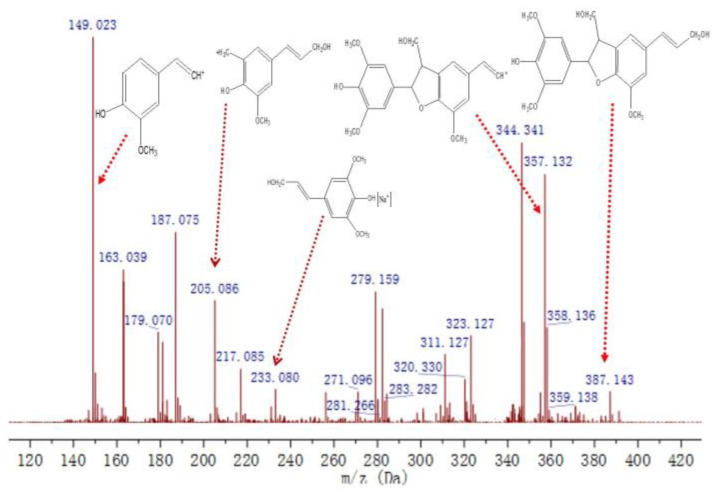
Mass spectrum of the compound GS_1_.

Figure 14 shows the mass spectral information of GS_1_. The *m*/*z* 205.086 signal was a typical fragment of β-5 dimer after the cleavage of the coumaran ring. The signal at *m*/*z* 233.080 was formed by the capture of Na^+^ by the sinapyl alcohol monomer. The ion peak at *m*/*z* 387.143 was derived from GS-type β-5 dimer with a detailed structure of ((β-5), γ-CH_2_OH, γ′-CH_2_OH). The signal at *m*/*z* 357.132 occurred due to the loss of the gamma position -CH_2_OH from the fragment of *m*/*z* 387.143, indicating that the -CH_2_OH at the gamma position is relatively easily lost, which again confirms the structure of GS_1_.

**Figure 15:**
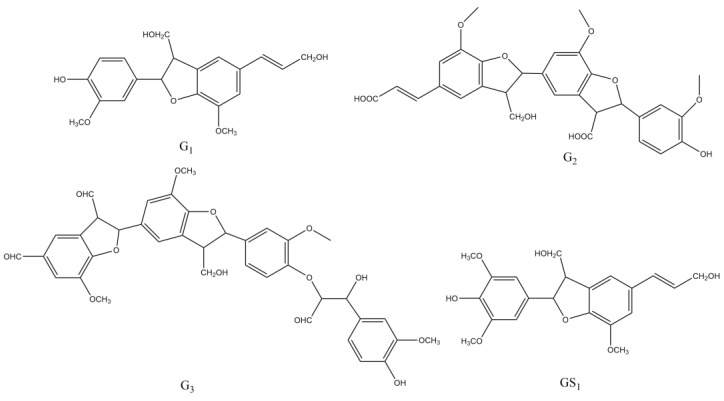
Chemical structure of the compound G_1_, G_2_, G_3_, and GS_1_ with significant antibacterial effects.

According to the analyses of mass spectra, the structures of the main bioactive compounds are shown in Figure 15. Compound G_1_, G_2_, G_3_, and GS_1_, which have strong inhibitory effects on *E. coli* and *S. aureus* are demonstrated above, and all contain β-5 structures. Differences in the antibacterial activities of these compounds mainly depended on the degree of polymerisation. The G_1_ is a G-type dimer (β-5, γ-CH_2_OH, γ′-CH_2_OH), and the G_2_ is a G-type trimer ((β-5)(β-5), γ-COOH, γ′-CHOH, γ″-COOH), while the G_3_ is a G-type tetramer ((β-O-4)(β-5)(β-5), α-OH, γ-CHO, γ′-CH_2_OH, γ″-CHO, α‴-CHO). Thus, as the molecular weight increased, the antibacterial activity of the compound was reduced to a certain extent. Xie et al. [43] found that the total phenol content which was related with antibacterial properties of DHPs decrease with the increase in their molecular weights. On the other hand, as compared with higher molecular weight substances, substances with lower molecular weight can easily penetrate the cell membrane of bacteria to have a better antibacterial effect. This result explains the phenomenon well, and some other researchers have similar findings [44,45,46,47].

The GS_1_ compound has an additional methoxy group at the fifth position of the S-type monomer compared to the G_1_, but the antibacterial activity of GS_1_ was significantly weaker than that of the G_1_. The syringyl structure reduced the antibacterial properties of the substance. Shaikh et al. [48] synthesised 14 coumaran derivatives, most of which were active against *Mycobacterium tuberculosis* (H37Rv), and found that the MICs of some compounds for *S. aureus*, *Bacillus* sps., and *E. coli* strains were as low as 0.8–1.6 μg/mL, while the MICs for *Candida albicans*, *Aspergillus flavus*, *Aspergillus niger*, and *Aspergillus fumigatus* were as low as 0.4–6.25 μg/mL. Senioa et al. [49] prepared hydromethanolic extracts and infusions from air-dried and freeze-dried *Galium aparine* L. containing phenylcoumaran to detect biological activity, and found that the MICs of the sample against *E. coli* and *S. aureus* were 3.75–30 mg/mL and 1.85–15 mg/mL, respectively. Xie et al. [50] used isoeugenol as a precursor to synthesise a low molecular weight DHP catalysed by laccase. Its antibacterial performance may be due to the existence of its β-5 structure, which was also observed in the present study. Hattori et al. [51] fractionated of the methanolic extract of the aril of *Myristica fragrans* Houtt. Followed by microbial assay using the tube dilution technique, they found that two substances containing phenylcoumaran structure could inhibit bacterial glucosyltransferase and cause loss of bacterial adhesion, which resulted in good antibacterial properties. The remaining three substances without phenylcoumaran structure had not this ability, resulting in poor antibacterial properties. The present results were in good agreement with their findings. This provides a useful direction for the development of the antibacterial industry in the future.

## 3. Materials and Methods

### 3.1. Materials

**Figure 16:**
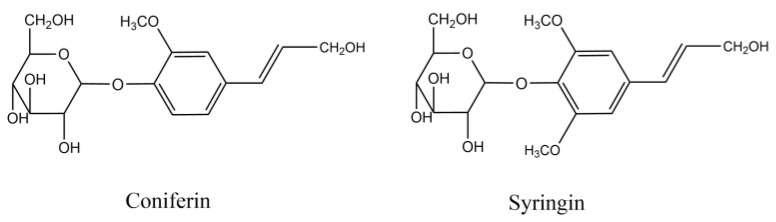
Chemical structure of coniferin and syringin.

Coniferin and syringin, as shown in Figure 16, were synthesised with vanillin and syringaldehyde, respectively [40,52]. β-Glucosidase was purchased from Sigma Co., Ltd. (Shanghai, China) and laccase (No.51003) from Novazyme Co., Ltd. (Tianjin, China). The other chemicals were of analytical grade, purchased from Sinopharm Chemical Reagent Co., Ltd. (Shanghai, China).

Gram-negative bacteria *E. coli* ATCC 25,922 and Gram-positive bacteria *S. aureus* CMCC (B) 26,003 were purchased from Shanghai Luwei Technology Co., Ltd. (Shanghai, China). Ordinary nutrient agar culture medium was purchased from Aobox Biotechnology Company (Beijing, China) and used for agar plates, which were autoclaved at 121 °C for 30 min.

### 3.2. Synthesis of DHP

**Figure 17:**
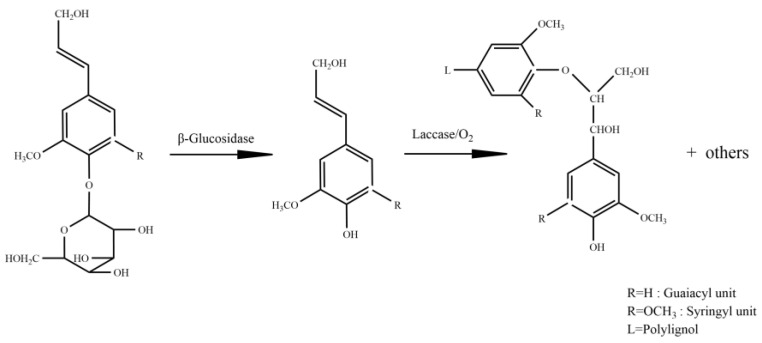
Chemical structure diagrams of DHP-G formation.

The chemical structure changes during the formation of DHP-G are shown in Figure 17. Coniferin (8.8 mmol) was dissolved in 0.2 mol/L sterile acetic acid/sodium acetate buffer solution (100 mL, pH 5.0), then β-Glucosidase (30 mg, 6.4 U/mg) and laccase (2 mL, 1093 IU/mL) were added with mixture. The reaction continued under sterile air filtered by activated carbon and the mixture reacted at 30 °C in a water bath for 30 min. The reaction was completed by adding 100 mL of distilled water after 30 min and heating in a water bath at 60 °C to end the reaction. The precipitated fraction was collected after centrifugation and washed with distilled water several times. After freeze-drying, the product was extracted with a mixture of dichloroethane/ethanol (2:1 *v*/*v*, 60 mL) for 6 h, and then centrifuged to collect the dissolved fraction [39]. The solvent was removed in vacuo to obtain purified DHP-G with a yield of 88.3%.

The synthesis of DHP-GS was consistent with the above steps, with raw material consisting of coniferin (4.0 mmol) and syringin (4.0 mmol), resulting in a yield of DHP-GS of 80.9%.

### 3.3. Classification of the DHP

**Figure 18:**
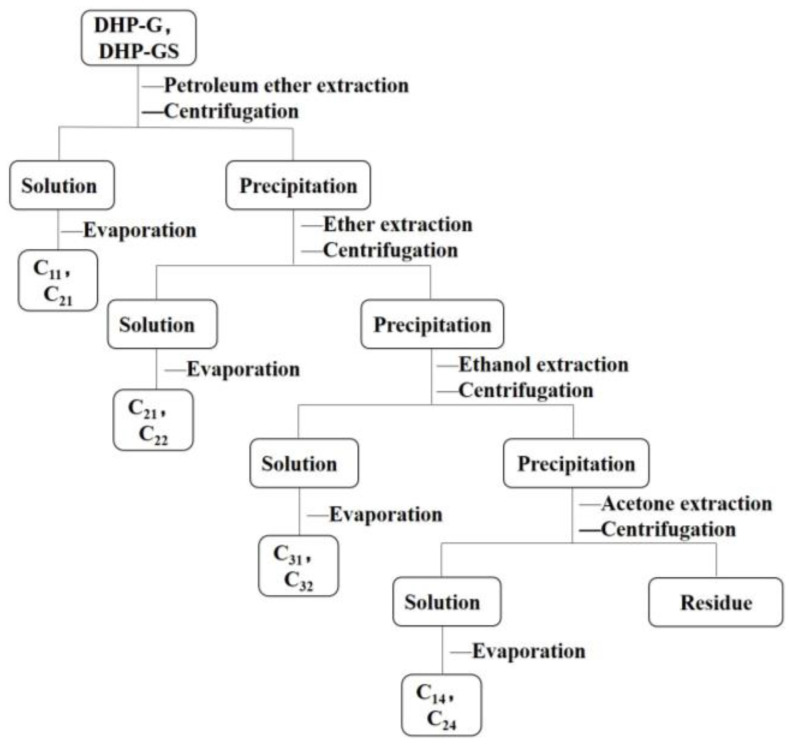
Classification flow chart of DHP-G and DHP-GS.

As shown in Figure 18, classification was conducted according to the polarity and solubility of different organic solvents and referring to the methods of Wang et al. and Li et al. [53,54]. Petroleum ether, ether, ethanol, and acetone were used to fractionate DHP-G and DHP-GS. The petroleum ether fraction, ether fraction, ethanol fraction, and acetone fraction were sequentially obtained from DHP-G, and the yields were 2.1%, 26.2%, 31.3%, and 7.8%, respectively. The petroleum ether, ether, ethanol, and acetone fractions were also obtained from DHP-GS, and the yields were 2.5%, 24.3%, 40.8%, and 9.7%, respectively.

### 3.4. ^13^C-NMR Measurement of DHP-G and DHP-GS

80 mg of DHP-G or DHP-GS sample was placed into a Φ 5 mm NMR tube and dissolved in 0.6 mL DMSO-*d*_6_ (0.6 mL). A 600-dd2 NMR spectrometer (DD2-600, Agilent Technologies, Santa Clara, CA, USA) was used to scan the solution at 150.83 MHz to obtain the corresponding ^13^C-NMR spectrum. The parameters of the instrument were: pulse delay: 2.5000 s, acquisition time: 0.9437 s, and scanning time: 6000 times.

### 3.5. Determination of Molecular Weight of the DHP Fractions

The relative molecular weight of each fraction of DHP was determined by size exclusion chromatography. Each DHP fraction (2 mg) was dissolved in N,N-dimethylformamide (DMF) (2 mL), filtered through a 0.22 μm microporous membrane, and then injected into a Shimadzu LC 20A gel permeation chromatograph (GPC) (LC 20A, Shimadzu, Kyoto, Japan). The separation column was a Shim-pack GPC-803D (300 mm × 8 mm) (803D, Shimadzu, Kyoto, Japan) and the mobile phase was DMF with a flow rate of 0.6 mL/min. The column temperature was 35 °C, and the injection volume was 25 μL. Polystyrene was used as the standard.

### 3.6. Purification of Ether Fraction of DHP with Preparative Column Chromatography

**Figure 19:**
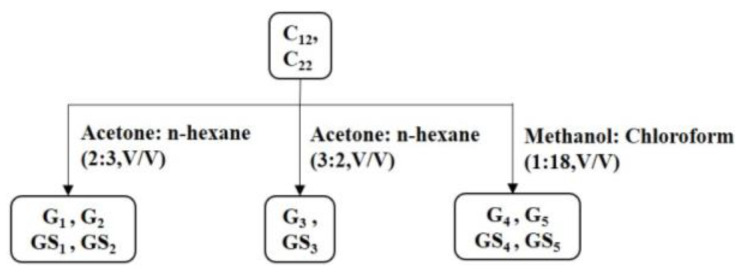
Purification process of DHP ether component.

As shown in Figure 19, referring to the methods of Tan et al. and Xiang et al. [55,56], the fractions C_12_ and C_22_ with high antibacterial activity were further purified by preparative column chromatography (Biuchi C-615, Buchi Lab Equipment, Flawil, Switzerland) and eluted with acetone/n-hexane (2:3 *v*/*v*), acetone/n-hexane (3:2 *v*/*v*), or methanol/chloroform (1:18 *v*/*v*). The yields of compounds G_1_ to G_5_ purified from C_12_ were 39.2%, 28.7%, 15.4%, 8.9%, and 7.8%, respectively. The yields of compound GS_1_ to GS_5_ purified from C_22_ were 43.5%, 26.9%, 12.0%, 10.4%, and 7.2%, respectively.

### 3.7. Determination of the Antibacterial Activity of DHP Fractions and Purified Compounds

The antibacterial activities of fractionated DHP components and purified compounds were determined by the filter paper agar diffusion method [57,58,59,60]. The *E. coli* or *S. aureus* suspension was diluted with a McFarland turbidity of 0.5 with sterile saline, and the bacterial concentration was approximately 1.5 × 10^8^ CFU/mL. On each agar plate, 200 µL diluted bacterial suspension were applied evenly. The sample was dissolved in dimethyl sulfoxide/normal saline (4:96 V/V, 0.1% Tween 80 as dispersant) to obtain a series of solutions with a concentration of 5 mg/mL. Dry sterile filter paper with a diameter of 6.00 mm was soaked in the above solution for 6 h, then removed and attached to the agar plate containing bacteria. Three filter paper discs were on each dish. The corresponding DHP sample solution (10 µL) was then added to the surface of the filter paper, which was incubated at 37 °C for 24 h. The diameter of the inhibition zone around the filter paper was observed and determined and the average value was calculated.

The minimum inhibitory concentration (MIC) was determined by the test tube two-fold serial dilution method, with a slight improvement [61]. Several 18 × 180 mm test tubes were prepared, and the first tube with broth medium with a concentration of 4 mg/mL and a volume of 4 mL. Then, 2 mL from the first tube was removed to the second tube and 2 mL of the corresponding solvent was added. This operation was repeated for 10 tubes; note that the last tube should also be taken out 2 mL to remove the corresponding solvent. Then, 50 μL of the diluted bacterial solution was added to each test tube and placed in the incubator for 24 h. The corresponding concentrations of non-turbid and turbid in adjacent test tubes were recorded. A series of gradient samples was prepared between the above concentrations, and bacteria solutions were added. The above steps were repeated three times to obtain more accurate MIC data and the average was taken.

### 3.8. Mass Spectrometry Analysis of the Structure of the Purified DHP Compounds

The molecular weights of compounds G_1_, G_2_, G_3_, and GS_1_ were determined by high-performance benchtop quadrupole trap atmospheric pressure chemical ionisation mass spectrometry (APCI-MS) (Q Exactive HR MS, Thermo Fisher Scientific, Waltham, MA, USA) [62,63,64]. The scanning range of the ion source was *m*/*z* 70–1050. Nitrogen was used as a drying gas and the flow rate was 45 mL/min.

## 4. Conclusions

1. DHP-G and DHP-GS were synthesised by laccase-catalysed dehydrogenation polymerisation. The ^13^C-NMR spectra showed that both DHP-G and DHP-GS contained β-O-4, β-5, β-β, β-1, and 5-5 substructures.
2. The ether-soluble fractions C_12_ of DHP-G and C_22_ of DHP-GS had strong antibacterial activities against *E. coli* and *S. aureus*.
3. The antibacterial activities of compounds GS_1_–GS_5_ purified from the C_22_ fraction and compounds G_1_–G_5_ separated from the C_12_ fraction were stronger against *E. coli* than *S. aureus*. The separated compounds G_1_, G_2_, G_3_, and GS_1_ had strong inhibitory effects against *E. coli* and *S. aureus*.
4. According to the results of APCI-MS, compound G_1_ was a (β-5) G-type dimer, while compound G_2_ was a (β-5) (β-5) G-type trimer, and G3 was a (β-O-4) (β-5) (β-5) G-type tetramer. GS_1_ was a (β-5) GS-type dimer.
5. Higher molecular weight and the introduction of syringyl units reduced the antibacterial activity. The antibacterial activity of G_1_, which had the best antibacterial activity, is attributable to the β-5-type connected phenylcoumaran dimer.

## Figures and Tables

**Table 1:** Average molecular weight of DHP fractions.

DHP Fractions	M_w_	M_n_	PDI
C_11_	289	192	1.51
C_12_	619	387	1.60
C_13_	1527	988	1.55
C_14_	2846	1923	1.48
C_21_	293	181	1.62
C_22_	677	462	1.47
C_23_	1478	860	1.72
C_24_	2642	1794	1.47

Legend: M_w_: weight average molecular weight; M_n_: number average molecular weight; PDI: polymer dispersity index; C_11_: petroleum ether-extracted fraction from DHP-G; C_12_: ether-extracted fraction from DHP-G; C_13_: ethanol-extracted fraction from DHP-G; C_14_: acetone-extracted fraction from DHP-G; C_21_: petroleum ether-extracted fraction from DHP-GS; C_22_: ether-extracted fraction from DHP-GS; C_23_: ethanol-extracted fraction from DHP-GS; C_24_: acetone-extracted fraction from DHP-GS.

## Data Availability

The data presented in this study are available in the manuscript.

## Sample Availability

Samples of the compounds are not available from the authors.
